# Synthesis of CuInS_2_ quantum dots using polyetheramine as solvent

**DOI:** 10.1186/s11671-015-0789-3

**Published:** 2015-03-12

**Authors:** Shih-Chang Shei, Wen-Jui Chiang, Shoou-Jinn Chang

**Affiliations:** Department of Electrical Engineering, National University of Tainan, Tainan, 70005 Taiwan; Institute of Microelectronics and Department of Electrical Engineering, Advanced Optoelectronic Technology Center, Center for Micro/Nano Science and Technology, National Cheng Kung University, Tainan, 70101 Taiwan

**Keywords:** CuInS_2_ quantum dots, Polyetheramine, Solvent, Photoluminescence

## Abstract

This paper presents a facile solvothermal method of synthesizing copper indium sulfide (CuInS_2_) quantum dots (QDs) via a non-coordinated system using polyetheramine as a solvent. The structural and optical properties of the resulting CuInS_2_ QDs were investigated using composition analysis, absorption spectroscopy, and emission spectroscopy. We employed molar ratios of I, III, and VI group elements to control the structure of CuInS_2_ QDs. An excess of group VI elements facilitated precipitation, whereas an excess of group I elements resulted in CuInS_2_ QDs with high photoluminescence quantum yield. The emission wavelength and photoluminescence quantum yield could also be modulated by controlling the composition ratio of Cu and In in the injection stock solution. An increase in the portion of S shifted the emission wavelength of the QDs to a shorter wavelength and increased the photoluminescence quantum yield. Our results demonstrate that the band gap of the CuInS_2_ QDs is tunable with size as well as the composition of the reactant. The photoluminescence quantum yield of the CuInS_2_ QDs ranged between 0.7% and 8.8% at 250°C. We also determined some important physical parameters such as the band gaps and energy levels of this system, which are crucial for the application of CuInS_2_ nanocrystals.

## Background

Nanoparticles have attracted considerable attention due to their unique properties and applicability in biomedicine [1-4], renewable energy [5-9], and optical devices [10-13]. The ability to control the optical properties of semiconducting nanoparticles, otherwise referred to as quantum dots (QDs), has prompted a great deal of research in bioimaging [14,15] and opto-electronic devices [16-19]. Cadmium-based QDs, including CdSe and CdTe, have been widely examined for their excellent physical properties; however, the toxicity of cadmium limits their applicability. QDs from the periodic groups III-V [20-24] and I-III-VI [19,25-27], referred to as cadmium-free QDs, have been proposed as an alternative by many research groups. In fact, InP/ZnS core/shell QDs, which are representative of III-V QDs, have been extensively studied and are close to commercialization. Other I-III-VI QDs, including copper indium sulfide (CuInS_2_) (CIS), CuInSe_2_ (CISe), AgInS_2_ (AIS), and AgInSe_2_ (AISe), have also been widely studied [25-30]. According to Peng et al. [29], the photoluminescence of the CIS core cannot be maintained for more than 4 days and the quantum yield is <4%. Thus, type-I core/shell structures, such as the CIS/ZnS were developed to enhance the stability and quantum yield of CIS QDs. Zinc ethylxanthate and zinc diethyldithiocarbamate have been used for ZnS shell growth on a CIS core, and interestingly, this has led to a blue shift in photoluminescence [28,31]. This phenomenon differs considerably from that of the general type-I core/shell structures, which show a red shift in emissions with an increase in the thickness of the particle shell. However, the explanation that has been posited is not convincing, despite the strong suggestion of surface reconstruction by Reiss et al. and the inter-diffusion of Zn atoms by Pons et al. [27,29]. Their findings suggest that cation exchange may explain the blue shift [31]. Since cation exchange was first reported, a number of studies have reported interesting results from a wide range of nanoparticles. According to these reports, the original shape and anion sublattice remain intact during the exchange of cations throughout the reaction. This paper presents a facile solvothermal method of synthesizing CuInS_2_ quantum dots (QDs) via a non-coordinated system using polyetheramine as a solvent. We demonstrated that this is a more effective way to produce monodisperse nanocrystals. Thus, a copper-thiol solution was injected into the solution, which included an In precursor. Our results give a clear indication that the blue shift in photoluminescence is caused by cation exchange.

## Methods

Indium (III) chloride (InCl_3_ · 3H_2_O, 99.99%), copper (II) chloride (CuCl_2_ · 2H_2_O, 99.99%), sulfur (S_2_, 99.99%), and D400 were purchased from Sigma-Aldrich (St. Louis, MO, USA). For the preparation of CuInS_2_ QDs, InCl_3_ · 3H_2_O (0.1 mol), CuCl_2_ · 2H_2_O (0.1 mol), sulfur (0.1 mol), and ODE (200 ml) were loaded into a 500-ml three-necked flask. The solution was degassed at 100°C for 1 h. In the first case, the temperature was gradually increased to 150°C, 200°C, or 250°C over a period of 8 h under N_2_ atmosphere. In the second case, the temperature was maintained at 250°C and the reaction time was varied between 1 and 8 h under an N_2_ atmosphere without stirring. It should be noted that extending the growth to beyond 8 h resulted in severe agglomeration of the QDs. In the third case, CIS QDs were synthesized using various ratios of Cu/In (/1, 3/4, and 1/2), which were prepared by fixing the amount of In precursor and varying the amount of Cu precursor. For the synthesis of CIS QDs with a Cu/In ration of 1/1 (i.e., 0.5 mmol of CuCl_2_ or 0.5 mmol of InCl_3_), we loaded 200 mL of D400 into 500 mL of a three-necked flask. The reaction mixture was then degassed during heating to 100°C and backfilled with N_2_ followed by further heating to 250°C over a period of 8 h. CIS core QDs were then allowed to grow for 5 min at that temperature. CIS QDs with Cu/In ratios of 3/4 and 1/2 were synthesized in precisely the same manner except that we employed 0.75 and 0. 5 mmol of CuI, respectively. After cooling the resulting CIS QD crude solution to room temperature, as-reacted CIS core QDs were precipitated with an excess of ethanol, purified repeatedly with a chloroform/ethanol solvent by centrifugation (8,000 rpm/30 min), and dispersed in chloroform. For characterization, the CIS core particles were purified by precipitation in an excess of acetone and dispersed in alcohol.

Ultraviolet-visible (UV-vis) and photoluminescence (PL) spectra were obtained using a Shimadzu UV-2450 UV-vis spectrophotometer (Shimadzu, Kyoto, Japan) and a Cary Eclipse (Varian Medical Systems, Palo Alto, CA, USA) fluorescence spectrophotometer, respectively. The room-temperature PL quantum yields (QYs) of nanocrystals (NCs) were determined by comparing the integrated emissions of the NC samples in solution with those of a fluorescent dye (rhodamine 6G in ethanol or rhodamine 101 in 0.01% HCl ethanol solution) with identical optical density. The known QY of the NCs in solution was used to measure the PL efficiency of other NCs by comparing their integrated emissions. Fluorescence lifetime was determined using an Edinburgh FL 900 single-photon counting system (Edinburgh Instruments Ltd., Livingston, West Lothian, UK) equipped with a Hamamatsu C8898 ps light pulser (Hamamatsu, Hamamatsu City, Shizuoka Pref., Japan). Excitation light was obtained using a 441-nm laser light with the luminescence time range set to 200 ns. Data were analyzed using a non-linear least squares fitting program, with deconvolution of the exciting pulse at approximately 200 ps. Transmission electron microscopy (TEM) was performed by depositing the NCs from dilute toluene solutions onto copper grids with a carbon support by slowly evaporating the solvent in air at room temperature. TEM images were acquired using a JEOL JEM-1400 transmission electron microscope (JEOL Ltd., Akishima-shi, Japan) operating at an acceleration voltage of 120 kV. Powder X-ray diffraction (XRD) measurements were obtained under wide-angle X-ray scattering using a Siemens D5005 X-ray powder diffractometer (Siemens, Munich, Germany) equipped with graphite-monochromatized Cu Kr radiation (λ *=* 1.54178 Å). XRD samples were prepared by depositing NC powder on a Si (100) wafer.

## Results and discussion

**Figure 1:**
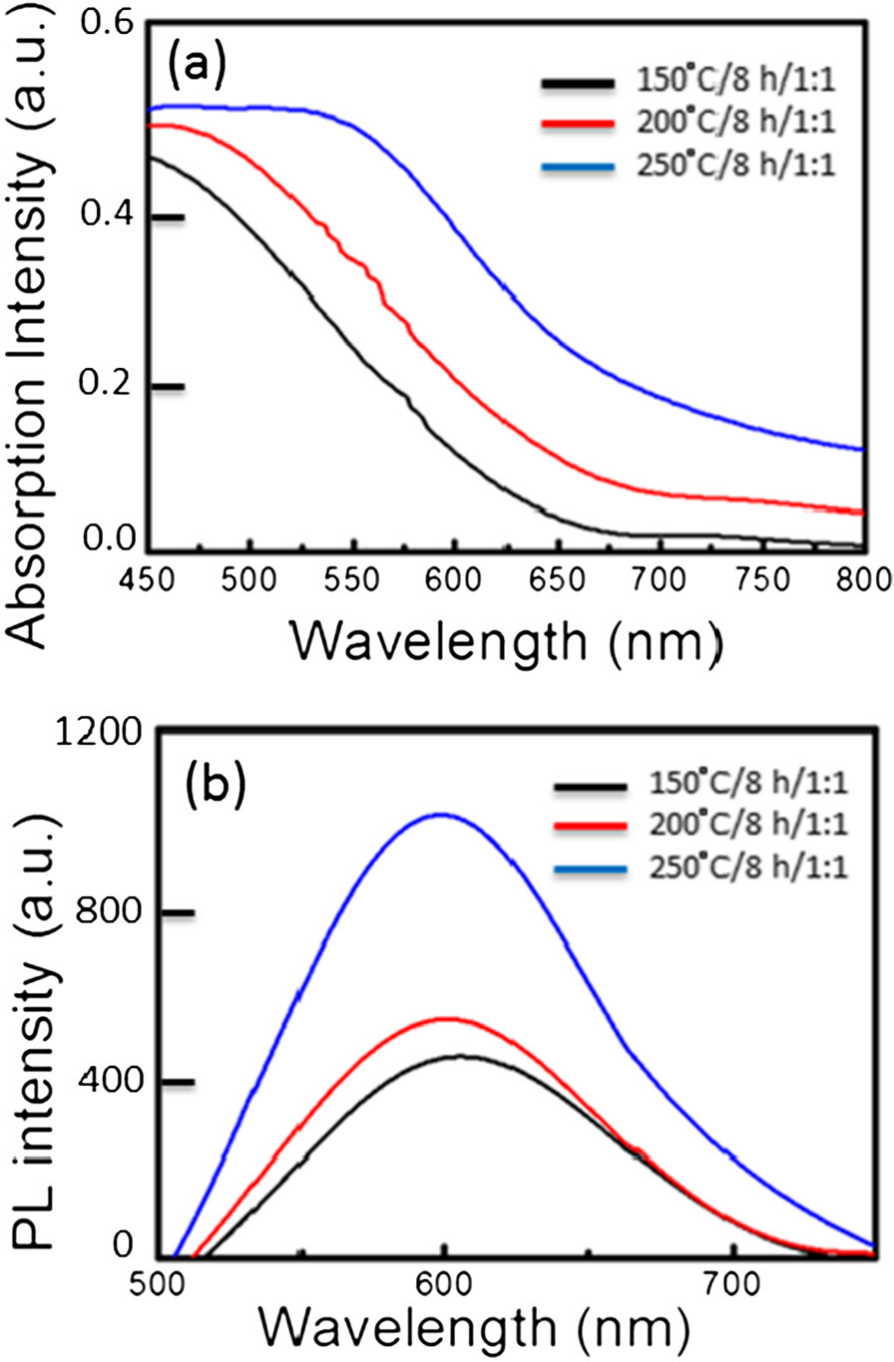
**Absorption spectra (a) and photoluminescence spectra (b) of the CIS QDs.**

CuInS_2_ QDs were synthesized using CuCl_2_ · 2H_2_O, InCl_3_ · 3H_2_O, and sulfur powder as precursors. Figure 1a presents the absorption spectra of CIS QDs with different solvothermal growth temperatures of 150°C, 200°C, and 250°C. The QDs grown at 250°C exhibited absorption at longer wavelengths, which are different from those grown at 150°C due to a reduction in the quantum confinement effect associated with QDs of larger size. CIS QDs grown at 150°C presented a blue shift in the absorption spectra of the resulting CIS QDs, as shown in Figure 1b. After increasing the growth temperature, the absorption features of CIS QDs became less pronounced; however, we observed a shift in the onset of absorption by CIS QDs to a longer wavelength. It should be noted that the optical band gaps of CIS QDs grown at 150°C, 200°C, and 250°C were estimated at 2.19, 1.82, and 1.65 eV, respectively. A similar narrowing in the band gap of CIS QDs with increased temperature has been attributed to an increase in the effective size of the QD core [9,11]. The emission QY values, calculated at an excitation wavelength of 450 nm, were as follows: 150°C-CIS QDs (3.7%), 200°C-CIS QDs (5.0%), and 250°C-CIS QDs (8.8%). As expected, the enhanced photoluminescence peaks appeared following an increase in the growth temperature from 150°C to 250°C. No changes were observed below 150°C. Furthermore, the full width at half maximum (FWHM) of emissions dropped was slightly from 120 to 105 nm. From our measurements, the growth temperature of 250°C is best for PL intensity.

**Figure 2:**
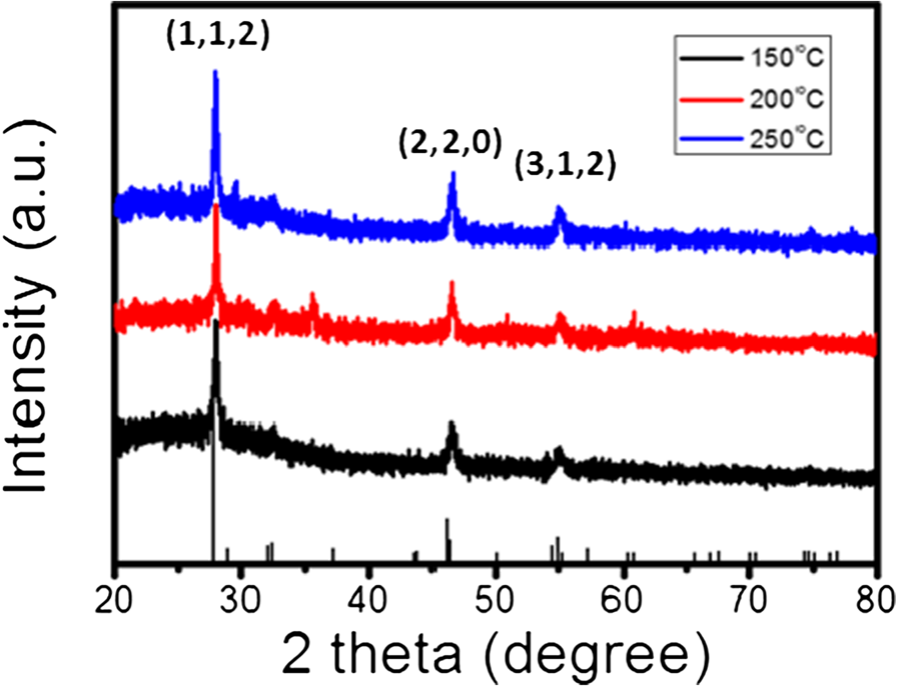
**XRD patterns of CIS QDs produced at various reaction temperatures.**

**Figure 3:**
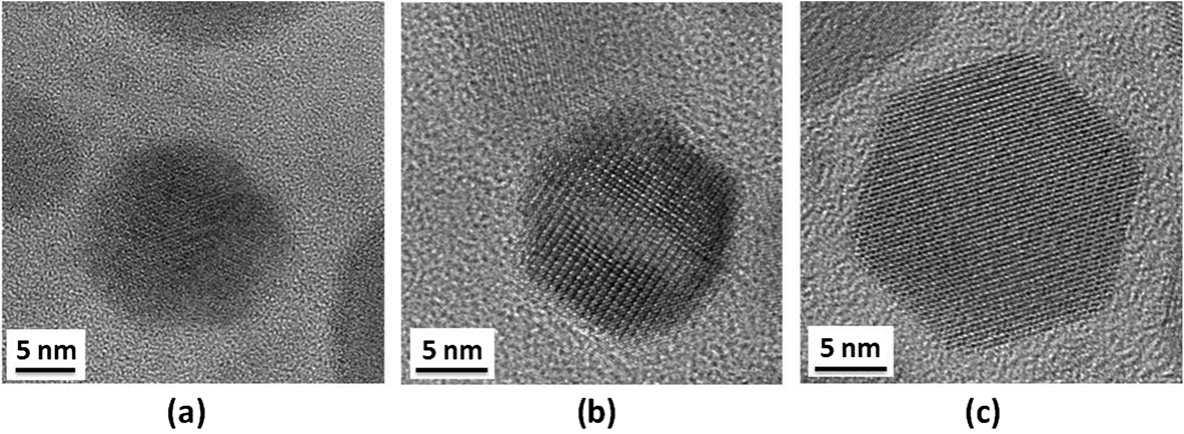
**TEM images of CIS QDs synthesized at temperatures of (a) 150°C, (b) 200°C, and (c) 250°C.**

Figure 2 presents changes in the powder XRD pattern obtained during the experiment on temperature dependence. Figure 2 shows XRD spectra measured from the CIS cores synthesized in this study. It can be seen that all these CIS cores exhibited three noticeable peaks which correspond to the (112), (220), and (312) reflection planes of the thermodynamically stable chalcopyrite tetragonal structure of CuInS_2_ phase (Joint Committee on Powder Diffraction Standards (JCPDS): 65-2732). The size of the crystal domain was estimated from the FWHM of the (112) peak using the Scherrer equation [19]:

1 $$ D=\frac{0.9\lambda }{b \cos \theta } $$

where D is the diameter of the crystallites forming the film, λ is the wavelength of the Cu Kα line, b is the FWHM in radians, and θ is the Bragg’s angle. Using the Debye-Scherrer equation, it was found that the average sizes of the CIS cores synthesized at 150°C, 200°C, and 250°C were 9.82, 10.41, and 13.68 nm, respectively. It was also found that XRD peak intensities increased with the synthesis temperature. This seems to suggest that CIS cores synthesized at higher temperatures could provide a better crystal quality. As shown in the TEM images in Figure 3, the sizes of the CIS QDs were about 10, 11, and 13 nm at solvothermal growth temperatures of 150°C, 200°C, and 250°C, respectively. The trend of TEM images is similar to the results of XRD measurements. In addition, their corresponding standard deviations of the size distribution are 0.98, 1.12, and 1.21 nm, respectively. The size of the CIS QDs increased with an increase in growth temperature, which is consistent with the XRD and PL data, as shown in Figures 1 and 2. Since the shape of the QD seems not circular, we cannot control of the QD shape regarding to different growth conditions. But, the compositions of the QD were measured and shown as Cu:In:S = 25.9:24.8:49.3.

**Figure 4:**
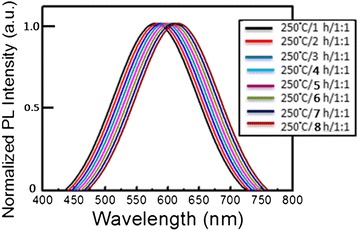
**Normalized photoluminescence spectra of the CIS QDs.**

**Figure 5:**
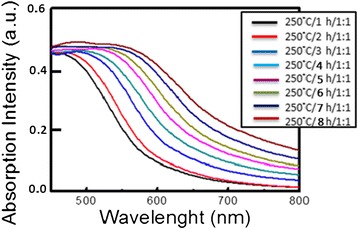
**Absorption spectra of CIS QDs synthesized under various reaction times.**

**Figure 6:**
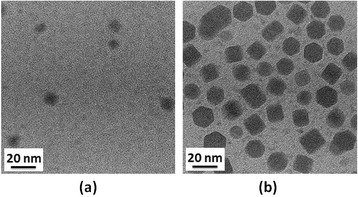
**TEM images of CIS QDs synthesized under reaction times of (a) 1 and (b) 8 h.**

Figure 4 presents the PL emission spectra of CIS QDs as a function of solvothermal growth time. CIS QDs were solvothermally synthesized at a fixed temperature of 150°C and Cu/In molar ratios of 1/1 for durations of 1, 2, 3, 4, 5, 6, 7, and 8 h, respectively. All CIS QDs presented orange color emissions (550 to 600 nm) with the peak wavelength slightly red-shifted in cases of longer growth time. The bandwidth (FWHM) of the emission of all CIS QDs fell within the range 98 to 105 nm, showing a tendency to form a broader emission band with longer growth time. Figure 5 presents the absorption spectra of CIS QDs grown at a fixed temperature of 150°C and Cu/In molar ratios of 1/1 for durations of 1, 2, 3, 4, 5, 6, 7, and 8 h, respectively. The absorption spectra gradually shifted to longer wavelengths with reaction time due to the growth of particles. Among various absorption spectrum, there is a long tail for the absorption profile due to the defect states (donors and acceptors) within the band gap. As reported in previous studies, distinct excitonic absorption features were not observed in the present CIS QDs. Such unresolved absorption features have been attributed to individual as well as combined factors, including a broad size distribution, unequal composition distribution, and the unique electronic properties of CIS QDs. The radiative recombination of excited electron-hole pairs in these kinds of QDs is associated with defect states (donors and acceptors) within the band gap, referred to as donor-acceptor pair (DAP) recombination [20-24]. The fact that many types of donor and acceptor sites exist suggests that the above defect energy levels may vary somewhat with QD size. Specifically, this would entail a shift in the donor and acceptor levels toward the edges of the conduction band (CB) and valence band (VB). Thus, it can be stated that a wide QD size distribution in the CIS QD ensemble is at least one of the reasons for the unresolved absorption peaks appearing in Figure 5. The normalized PL emission spectra of QDs are also presented in Figure 4, where a similar red shift in the absorption spectra with a decrease in QD size can be observed. The figure specifically shows a shift in peak emission wavelength from 550 nm (for 1-h QD) to 600 nm (8-h QD). This kind of shift in size-dependent emission toward the red in size sorted QD fractions may provide additional support for the supposition that the energy spacing between donor and acceptor levels increases with an increase in the size of the QDs. Broadband emissions are characteristic of DAP recombination resulting from a combination of strong electron-phonon interaction and a wide donor–acceptor distance distribution [19], which can be ascribed to size and/or compositional inhomogeneity in the QDs. Therefore, the nature of DAP recombination is likely the sole factor responsible for the broadness of CIS QD emissions, if the chemical composition of individual QDs is assumed to be homogeneous. Figure 6 presents TEM images of CIS QDs grown for 1 and 8 h, both of which are not monodispersed. The sizes of QDs produced through a 1-h reaction were distributed in the range of 2 to 10 nm, while those produced over a period of 8 h grew only slightly more to 8 to 16 nm, which is the most appropriate shell phase for the surface passivation of chalcopyrite I-III-VI QDs.

**Figure 7:**
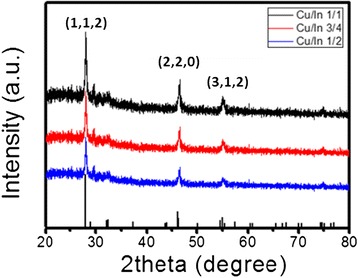
**XRD patterns of CIS QDs synthesized with Cu/In ratios of 1/1, 3/4, and 1/2.**

**Figure 8:**
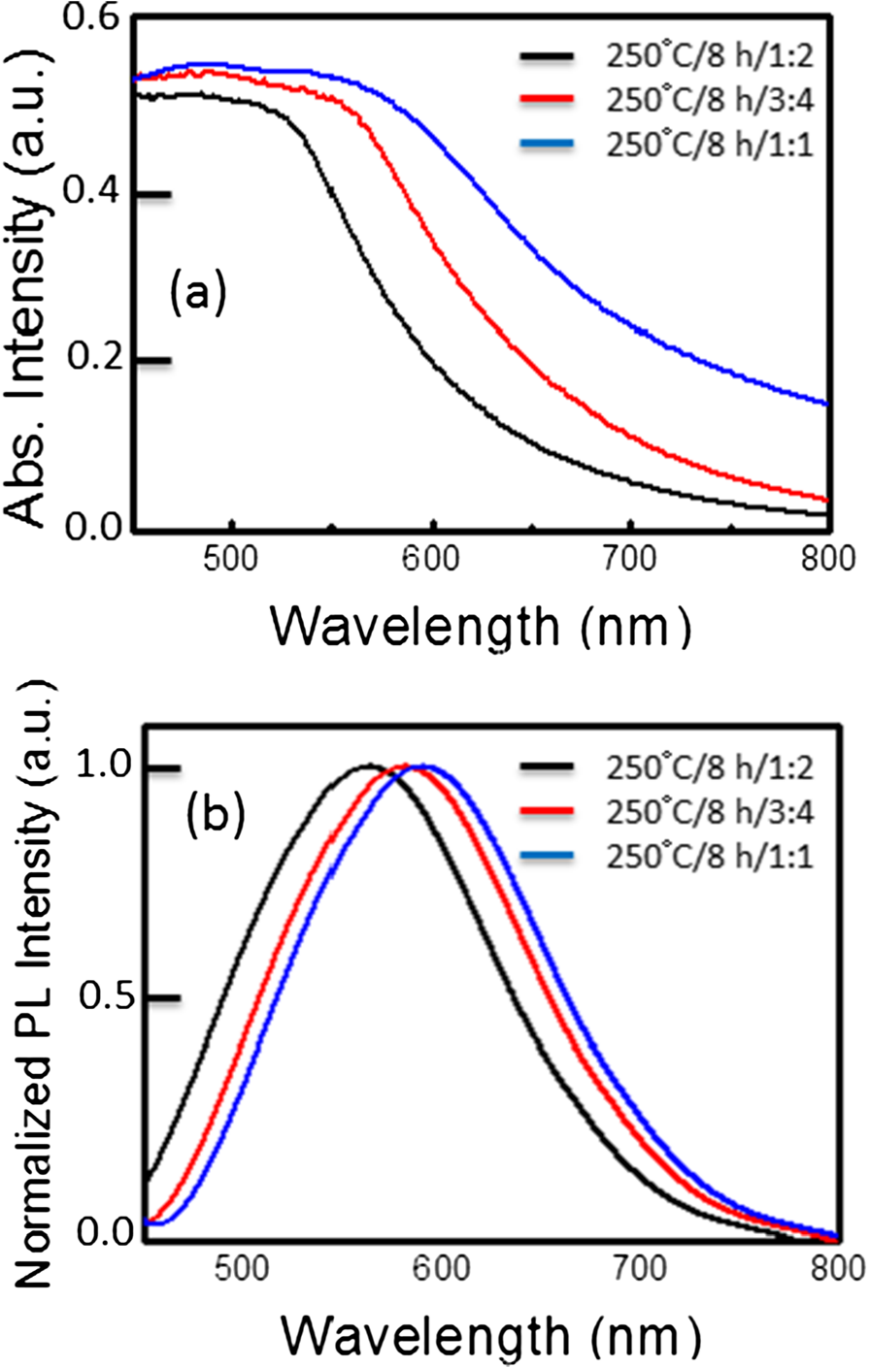
**Absorption (a) and normalized PL emission spectra (b) of CIS QDs synthesized under Cu/In ratios of 1/1, 3/4, and 1/2.**

We also examined the CIS QDs synthesized starting solution of various concentrations with different Cu/In molar ratios and at a fixed temperature of 150°C and for durations of 8 h. Figure 7 presents the XRD patterns of QDs with a CIS core produced at various Cu/In molar ratios of 1/1, 3/4, and 1/2. No discernible difference in reflection peak angle was observed, despite a relatively large variation in Cu content. Three distinct reflection peaks with 2θ values of 28.0°, 46.5°, and 54.9° were well indexed along the (112), (220), and (312) planes, respectively, of a known tetragonal chalcopyrite structure of the CuInS_2_ phase [30,31]. These XRD results are consistent with those in previous studies, in which the XRD patterns of highly off-stoichiometric (Cu/In ratio of 1/2) CIS QDs were the same as those of the stoichiometric QDs (Cu/In ratio of 1/1) [5]. The generation of a metastable In-rich CIS phase, such as CuIn_3_S_5_, could be expected under the Cu-deficient synthesis; however, the identification of such a metastable phase is challenging in practice, due to its structural similarity to the CuInS_2_ phase and XRD peak broadening. Nevertheless, according to Raman spectroscopic results obtained from CIS QDs with various degrees of Cu deficiency provided by Uehara et al. [5], the off-stoichiometric CIS QDs in this study, with Cu/In ratios of 3/4 and 1/2, are believed to possess the same chalcopyrite framework as stoichiometric QDs, despite the fact that such Cu-deficient QDs are likely to include Cu-related defects concentrated at high density (e.g., Cu vacancy and In interstitial at the Cu site). Figure 7 presents a TEM image of representative CIS QDs with a Cu/In ratio of 1/1, with widely dispersed size concentration in the range of 10 to 20 nm. Figure 8a presents the absorption spectra of CIS QDs with various Cu/In ratios, showing a blue shift in absorption far more pronounced than that observed in Cu-deficient CIS QDs. As mentioned previously in the context of the TEM results, this blue shift is not related to size variation. This variation in Cu/In composition-dependent band gap in the CIS QDs is consistent with previous reports, which generally attributed this effect to a lowering of the valence band due to the weakened repulsion between Cu d and S p orbitals in Cu-deficient material, ultimately leading to a widening of the band gap [26-28]. As shown in the normalized emission spectra and fluorescence images of CIS QDs in Figure 8b, all core QDs produced emissions in the orange-red region (with peak wavelengths of 600 nm for Cu/In = 1/1 and 580 nm for Cu/In = 1/2) with broad emission bandwidths of 110 to 125 nm. The systematic blue shift in emissions observed in the QDs with greater Cu deficiency is likely associated with a widening in the band gap, as described above. A large Stokes shift in emissions versus absorption wavelength up to approximately 650 meV implies that the radiative decay is unlikely to stem from carrier recombination between quantized electron-hole levels, but rather is associated with internal and/or surface defect sites that serve as intragap states. Nonetheless, the accurate assignment of electron-hole recombination channels in CIS QDs remains ambiguous. A commonly accepted transition mechanism is the so-called DAP recombination [22,27,29,30] in which In_Cu_ (In substituted at the Cu site) and/or V_S_ (S vacancy) probably act as donor states with *V*_Cu_ (Cu vacancy) as an acceptor state [29]. The explanation provided by Li et al. [26] regarding the alternative carrier recombination between the quantized conduction band minimum and defect (acceptor) trap level in CuInSe QDs is also persuasive. The broad bandwidth of emissions is characteristic of defect-related radiative transitions, as in the case of CIS QDs. Inhomogeneity in the size of QDs could lead to the broadening of emissions. However, previous findings suggest that even in a comparison of size-selective precipitated CIS QDs, an improvement in size inhomogeneity rarely alters the emission bandwidth [29]. The emission QY of CIS QDs with Cu/In ratios of 1/2, 3/4, and 1/1 were measured at 6.3%, 7.5%, and 8.8%, respectively. This increase in QY with more Cu-deficient QDs is in agreement with the results reported by Uehara and Nam et al. [5]. A higher density of Cu-related defect states through the formation of Cu-deficient QDs would increase the probability of carrier recombination, resulting in enhanced efficiency. From the above discussions, we conclude that the best growth condition is at the temperature of 250°C, reaction time of 8 h, and Cu/In ratios of 1/1 to obtain the highest PLQY of CIS QDs.

## Conclusions

This study synthesized highly luminescent CuInS_2_ QDs. The emission quantum yield (QY) of CIS QDs synthesized at 150°C, 200°C, and 250°C reached 3.7%, 5.0%, and 8.8%, respectively, when calculated at an excitation wavelength of 450 nm. As expected, an increase in the photoluminescence peaks was observed as the temperature was increased from 150°C to 250°C. In addition, QDs with CIS core were solvothermally prepared by varying the growth time. All of the CIS QDs exhibited a size-dependent red shift in emissions with longer growth time; however, the emission wavelengths (550 to 600 nm) fell deep within the red region with PL QYs of 0.7% to 8.8%. The radiative transition was described according to the DAP recombination of photo excited carriers resulting in a large Stokes shift and broad PL band. All CIS core QDs exhibited relatively red shift in emission. This red shift in emissions can be ascribed to a shift of the donor and acceptor levels associated with band gap widening as well as an increased probability of DAP recombination between closely spaced pairs. Finally, we characterized QDs with a CIS core at Cu/In molar ratios of 1/2, 3/4, and 1/1. A widened band gap in Cu-deficient CIS QDs led to a systematic increase in the blue shift in emissions according to the degree of Cu deficiency. CIS QDs exhibited broadband defect emissions with peak wavelengths of 560 to 600 nm and QY of 6.3% to 8.8%. The factor which will be the most important one to influence the PLQY is the reaction temperature. It was also found that XRD peak intensities and PLQY increased with the synthesis temperature. This seems to suggest that CIS cores synthesized at higher temperatures could provide a better crystal quality.
